# A New Species of River Dolphin from Brazil or: How Little Do We Know Our Biodiversity

**DOI:** 10.1371/journal.pone.0083623

**Published:** 2014-01-22

**Authors:** Tomas Hrbek, Vera Maria Ferreira da Silva, Nicole Dutra, Waleska Gravena, Anthony R. Martin, Izeni Pires Farias

**Affiliations:** 1 Laboratory of Evolution and Animal Genetics (LEGAL), Department of Biology, Institute of Biological Sciences, Federal University of Amazonas (UFAM), Manaus, AM, Brazil; 2 Laboratory of Aquatic Mammals (LMA), National Research Institute of the Amazon (INPA), Manaus, AM, Brazil; 3 Centre for Remote Environments, University of Dundee, Dundee, United Kingdom; Zoological Society of London, United Kingdom

## Abstract

True river dolphins are some of the rarest and most endangered of all vertebrates. They comprise relict evolutionary lineages of high taxonomic distinctness and conservation value, but are afforded little protection. We report the discovery of a new species of a river dolphin from the Araguaia River basin of Brazil, the first such discovery in nearly 100 years. The species is diagnosable by a series of molecular and morphological characters and diverged from its Amazonian sister taxon 2.08 million years ago. The estimated time of divergence corresponds to the separation of the Araguaia-Tocantins basin from the Amazon basin. This discovery highlights the immensity of the deficit in our knowledge of Neotropical biodiversity, as well as vulnerability of biodiversity to anthropogenic actions in an increasingly threatened landscape. We anticipate that this study will provide an impetus for the taxonomic and conservation reanalysis of other taxa shared between the Araguaia and Amazon aquatic ecosystems, as well as stimulate historical biogeographical analyses of the two basins.

## Introduction

**Figure 1:**
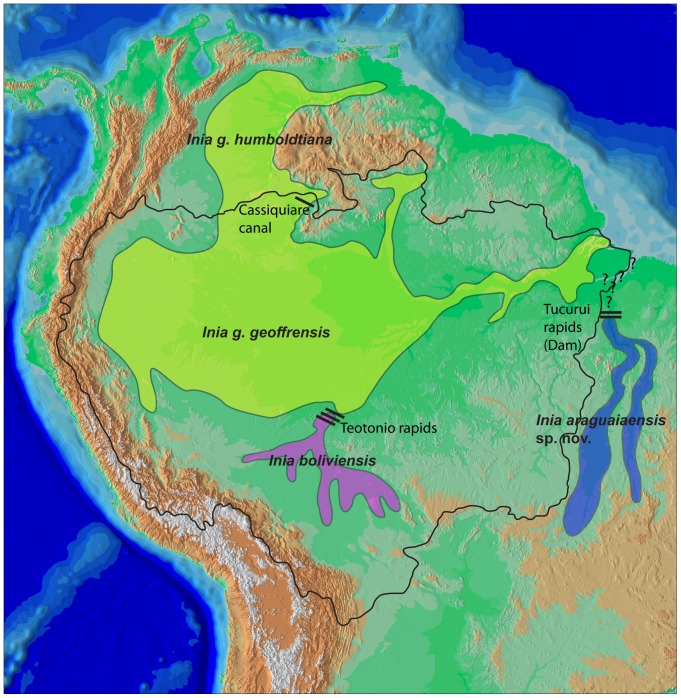
Distribution map of all known species and subspecies of *Inia*. Black outline denotes the limit of the Amazon basin. Question marks denote uncertainty as to which species occurs in the Tocantins River downstream of the Tucuruí dam which potentially delimits the distributions of *I. geoffrensis* and *I. araguaiaensis* sp. nov. Bars on the Madeira River represent a series of rapids that delimit the distribution of *I. geoffrensis* and *I. boliviensis*. The single bar on the northern limit of the Amazon basin represents the Casiquiare canal which connects the Amazon and Orinoco basins, and is thought to delimit the *I. g. humboldtiana* subspecies from *I. g. geoffrensis*.

True river dolphins of the genus *Inia* are widely distributed throughout the Amazon, Orinoco, and Araguaia-Tocantins basins (Fig. 1) [1]. The Araguaia-Tocantins River basin currently does not form part of the Amazon River basin [2], [3], the two basins having become disconnected although not completely isolated at the transition of the Pliocene to the Pleistocene [2]. Currently only a narrow canal west of Marajó Island links the Amazon River delta with the Pará River into which the Araguaia-Tocantins River drains. Connectivity between the Araguaia-Tocantins and Amazon basins is further restricted by a series of major rapids on the lower Tocantins River as it transits from the Brazilian Shield to the Amazonian lowland plain, the largest of which have been flooded in the process of creation of the Tucuruí Dam (03° 50′ 0.36″ S; 49° 38′ 15.55″ W). Major barriers such as waterfalls and rapids are thought to delimit *Inia* taxa as in the case of the upper Madeira River rapids which are thought to delimit *I. geoffrensis* and *I. boliviensis* [1], [4], [5]. Similarly, the rapids on the upper Orinoco and Negro Rivers together with the Cassiquiare Canal are thought to delimit the Orinoco and Amazon subspecies of *I. geoffrensis* [1], [4], [5]. The Araguaia-Tocantins River system is thus not only almost completely disconnected from the Amazon River basin, the portion of it that flows on the Brazilian Shield is isolated by rapids from the mouth of the river system and the Amazon River delta by a series of rapids. It is therefore possible that *Inia* individuals upstream of these rapids or even in the entire system represent a biological entity evolutionarily independent of *I. geoffrensis* in the Amazon basin. To test this hypothesis we analyzed a series of mitochondrial DNA, nuclear DNA and morphological characters of the two known species of *Inia* as well as individuals from the Araguaia River basin.

## Materials and Methods

### Ethics Statement

Samples were collected either from carcases, or from live animals using minimally invasive sampling procedures [6]. Protocol for handling and removing small quantities of cutaneous tissue samples from the caudal fluke of live animals was approved by the Committee on the Ethics of Animal Use (Comissão de Ética do Uso de Animais – CEUA) of National Research Institute of the Amazon (INPA).

### Field Sampling

Samples were obtained from animals in the Amazon-Solimões basin, the Bolivian sub-basin, and the Araguaia River. Samples were maintained in 95% ethanol and deposited in the official tissue collection (Coleção de Tecidos de Genética Animal – CGTA) of the Federal University of Amazonas and the tissue collection (Coleção de Tecidos) of the Instituto Nacional de Pesquisas da Amazônia. The licenses for collections were obtained through IBAMA/ICMBIO No. 11325-1 to IPF, No. 13462-1 to VMS, and No. 16741-2 to ND. In total we sampled 45 *I. boliviensis* from the Bolivian sub-basin, 44 *I. geoffrensis* from the Amazon-Solimões basin and 32 *Inia* sp. individuals from the Araguaia River.

### Nuclear Microsatellite DNA Amplification

Total genomic DNA was extracted from skin samples using Qiaquick blood and tissue kit (Hilsden, Germany) following the manufacturer’s protocol. Polymerase Chain Reaction (PCR) amplification was performed on total genomic DNA. Negative controls were run for all reactions.

To characterize *Inia* individuals, we used 7 microsatellite pairs (Ig2B1, Ig11B1, Ig8H1, Ig10E, Ig11D2, Ig3A1, Ig7F2) developed by Gravena *et al*. [7] for *I. geoffrensis*, and 3 microsatellite pairs (Ttr11, Ttr48, TtruAAT40) developed by Caldwell *et al*. [8] and Rosel *et al*. [9] for *Tursiops truncatus*. Genotyping was done according Gravena *et al*. [7] on the ABI 3130XL (Life Technologies) automatic sequencer. The program GeneMapper (Life Technologies) was used to extract raw fragment data, and infer fragment sizes.

### Mitochondrial DNA Amplification

The complete cytochrome *b* gene was amplified using standard PCR conditions with the primers IniaGluF.1 (5′-GACCAATGACATGAAAAATCATCG-3′) and IniaProR.1 (5′-CAGCTTTGGGTGTTGGTGGTGA-3′) developed for this study. The cytochrome oxidase I DNA barcode region was amplified using the C_VF1LFt1-C_VRLRt1 primer cocktail [10]. All the regions were amplified in a final volume of 15 µl and contained 7.6 µl of ddH_2_O, 1.2 of 25 mM MgCl_2_, 1.2 µl of 10 mM dNTPs, 1.5 µl of 10x buffer (750 mM Tris-HCl – pH 8.8 at 25°C, 200 mM (NH_4_)_2_SO_4_, 0.1% (v/v) Tween 20), 1.5 µl of each primer (2 µM), 0.3 µl of Taq DNA Polymerase (1 U/µl) and 1 µl of DNA (concentration varied between 10 ng and 30 ng). PCR reagents were obtained from Fermentas (Vilnius, Lithuania). PCR reaction consisted of 35 cycles of denaturation at 93°C for 60 seconds, primer annealing at 50°C for 40 seconds, and primer extension at 72°C for 90 seconds, followed by a final extension at 72°C for 5 minutes. PCR products were purified using EXO-SAP (Exonuclease – Shrimp Alcaline Phosphatase) following the manufacturer’s suggested protocol [11].

PCR products were evaluated on a 1% agarose gel, and then purified using the polyethylene glycol and ethanol precipitation protocol [12]. cytochrome *b* PCR products were sequenced using the primer IniaCytbF.1 (5′-CTACCCTGAGGACAAATATCATT-3′), IniaCytbF.2 (5′-CCCGACCTACTAGGAGACCC-3′), IniaCytbF.3 (5′-CCCGACTTACTAGGGGACCC-3′) and IniaCytbR.1 (5′-GAAGAAGCGTGTTAGTGTTGC-3′) developed for this study. cytochrome oxidase *c* I DNA barcode region PCR products were sequenced with M13F(−21) and M13R(−27) primers [13]. Cycle sequencing PCR followed manufacturer’s recommended protocol for BigDye sequencing kit (Life Technologies); primer annealing temperature was 50°C. Cycle sequencing reaction products were resolved on the ABI 3130XL (Life Technologies) automatic sequencer. Sequence products were edited, concatenated and aligned using the program Geneious v5.6.3 [14]. Sequences are deposited in Genbank under accession numbers KF657385– KF657432, KF657593– KF657640, KF802241– KF802276, KF802313– KF802348, KF802349– KF802380 and KF802381– KF802412.

### Population Genetic Characterization of Nuclear Microsatellite Data

The program ARLEQUIN version 3.5.1.2 [15] was used to test for linkage disequilibrium between pairs of loci, and to test for Hardy-Weinberg equilibrium. In cases of multiple comparisons, p-values were adjusted using the Holm method [16] implemented in the stats package of the software R 2.14.1 [17].

### Structure Analyses of Nuclear Microsatellite Data

The existence of biological groups is a necessary prerequisite for inferring the existence of a species under the Biological Species Concept (BSC) [18]. The microsatellite data were therefore analyzed in the program STRUCTURE version 2.3.3 [19], [20] with the goal of assigning individuals to groups, given a specific number groups (K). We used the ‘admixture’ and ‘correlated-allelic-frequencies’ models with a location prior. Assignment space was explored with 1,000,000 MCMC chains, preceded by 100,000 MCMC chains discarded as burn-in. Convergence was examined by viewing profiles of posterior probabilities, and values of α. Each analysis was repeated 20 times from a different randomly selected starting point, raw output was processed in Structure Harvester 0.6.92 [21]. The 20 independent runs were summarized in the program CLUMPP 1.1.2 [22], and results were visualized in the program DISTRUCT 1.1 [23]. The most likely number of biological groups (K) was inferred using Bayes’ Rule [24].

### Principal Components and Correspondence Analysis of Nuclear Microsatellite Data

The objective of this analysis was to reduce the dimensionality of the microsatellite data using the Ade4 package [25] in the software R 2.14.1 [17]. The first and second principal components were then plotted against each other, and the dispersion of points within each sample group was visualized by an ellipse encompassing a 67.53% density contour around each group representing the *Inia* from the Araguaia River, *I. geoffrensis* and *I. boliviensis*. Discrimination of the three groups of *Inia* was further assessed using the correspondence analysis implemented in the Ade4 package of R. Around each group an ellipse encompassing a 67.53% density contour was drawn.

### Population Aggregation Analysis and Phylogenetic Analysis of Mitochondrial DNA Data

Evolutionary lineages are diagnosed by discontinuities in character variation among lineages, and correspond to phylogenetic species. The existence of lineages is therefore a necessary and sufficient prerequisite for inferring the existence of a species under the different conceptualizations of the Phylogenetic Species Concept (PSC) [26]–[28]. The existence of lineages in a non tree-based context [26] was inferred using Population Aggregation Analysis performed at the level of an individual [29], [30], while the existence of lineages in a phylogenetic tree-based context [27], [28] was performed using Maximum Parsimony [31] and Bayesian Inference [32] analyses. Maximum Parsimony topology was estimated using PaupRat [33] implemented on the CIPRES Science Gateway website [34]. We used the TBR option to swap topologies, and ran 100 independent replicates. Bayesian Inference topology for molecular data was estimated in MrBayes 3.1.2 [32]; we ran two replicates, each with four independent chains for 10 million MCMC generations, sampling a topology every 10,000 generations. Searches were done using the General Time Reversible (GTR) model of molecular evolution [35] with probability of change from one nucleotide state to another assumed to follow a gamma distribution, and a portion of sites were assumed to be invariant. The GTR+G+I model was suggested as the most appropriate model of molecular evolution in jModeltest [36] using the Akaike Information Criterion (AIC) [37] to select among alternative models. To improve the accuracy of phylogenetic reconstruction [38], [39], we partitioned the data *a priori* by codon position. The GTR+G+I model of molecular evolution was applied to each codon position, and parameters were assumed to be uncorrelated between partitions. Convergence between the two independent MrBayes runs was assessed by comparing the posterior probability estimates for each analysis using a scatter-plot created by the *compare* command in AWTY [40]. Convergent topologies were combined for the calculation of posterior clade probabilities. Following Huelsenbeck *et al*. [41], the frequency of a clade among the sampled trees was interpreted as the posterior probability of that clade; the posterior probabilities are true probabilities under the assumed model of substitution [42]; thus we considered clades to be significantly supported when Bayesian posterior probabilities were ≥95% [43].

### Phylogenetic Dating Using Mitochondrial DNA Data

The objective was to estimate the divergence time of the three lineages of *Inia* from their respective sister taxa. To provide calibration points, we included taxa that resulted from diversification events for which a good fossil record exists, including taxa near the root of our phylogeny. We included additional taxa for the purpose of cross validation of our estimates with those published by Xiong *et al*. [44] based on complete mitochondrial genomes. We used prior age distributions so that they encompassed the entire period of the origination of the fossil as reported in literature, unless a more precise fossil age estimate was provided by one of the authors of the fossil literature. Calibrations were performed in the program BEAST 1.7.1 [45], with divergence times being lognormally distributed. Calibrations were chosen such that the youngest age of the distribution corresponded with the youngest fossil age estimate, and the oldest fossil estimate was encompassed within the 95% credible interval of the lognormal age distribution. Calibrations follow those used by Xiong *et al*. [44] with modifications of the 95% credible intervals to truly and exactly encompass the upper and lower fossil ages. The following four calibration points were used:

1. The divergence between Mysticeti (baleen whales) and Odontoceti (toothed whales) was calibrated using the earliest record of mysticetes from the Eocene/Oligocene boundary (see [46] for a review). There has been debate about when the Eocene/Oligocene transition occurred (38–33.5 Mya in [47]), and therefore we chose a lognormal age prior so that the earlier age corresponded to 33.5 Mya and the older 95% credible interval (CI) encompassed the beginning of the Late Eocene (40 Mya) (standard deviation = 1.068).
2. The age of the root of crown Odontoceti was calibrated using the earliest record of a physeterid (sperm whales) – the Late Oligocene *Ferecetotherium* [48]; see also [49]. The age prior was lognormally distributed so that the youngest age corresponded to 23.7 Mya, the end of the Late Oligocene, and the older 95% credible interval (CI) encompassed the beginning of the Late Oligocene (30 Mya) (standard deviation = 1.135).
3. The divergence between Iniidae (the Amazon River dolphins) and Pontoporidae (La Plata River dolphins) was calibrated using the earliest record of a pontoporid, the Middle Miocene *Brachydelphis* [50]. The age prior was lognormally distributed so that the youngest fossil age corresponded to 11.2 Mya and the older 95% CI encompassed the beginning of the Middle Miocene (16.6 Mya) (standard deviation = 1.223).
4. The divergence between Phocoenidae (porpoises) and Monodontidae (narwhals) was calibrated using the earliest record of a phocoenid, the Late Miocene *Salumiphocaena* [51]. This fossil is approximately 10–11 million years old [51], and therefore we chose a lognormal age prior with the younger age corresponding to 10 Mya and the older 95% CI encompassing the beginning of the Late Miocene (11.2 Mya) (standard deviation = 1.061).

The BEAST analysis consisted of 1×10^8^ generations with a random starting tree, the Yule speciation prior, uniform [0, 100] prior distribution for the GTR+G+I substitution rates, default values for remaining priors, random starting topology, and topologies were sampled every 10000 generations. To improve the accuracy of phylogenetic reconstruction [38], [39], [52], we partitioned the data *a priori* by codon position. The topologies were used to calculate posterior clade probabilities and divergence times. The reliability of the estimated divergence times was further assessed by calculating an expected substitution rate compatible with the observed divergence time.

### Cranial Measurement Analyses

Cranial measurements and counts follow da Silva [1] with data from da Silva [1] serving as a base for comparison. All measurements were standardized as described in da Silva [1] while counts were analyzed untransformed. Since only few individuals were available for morphological analyses, no statistical analyses were performed and only means and ranges of equivalent measurements and counts were compared to those taken from *I. geoffrensis* and *I. boliviensis*.

### Nomenclatural Acts

The electronic edition of this article conforms to the requirements of the amended International Code of Zoological Nomenclature, and hence the new names contained herein are available under that Code from the electronic edition of this article. This published work and the nomenclatural acts it contains have been registered in ZooBank, the online registration system for the ICZN. The ZooBank LSIDs (Life Science Identifiers) can be resolved and the associated information viewed through any standard web browser by appending the LSID to the prefix “http://zoobank.org/”. The LSID for this publication is: urn:lsid:zoobank.org:pub:2F417149-4E97-4245-BF83-A8FA06388BEB. The electronic edition of this work was published in a journal with an ISSN, and has been archived and is available from the following digital repositories: PubMed Central and LOCKSS.

## Results

We collected molecular data from 44, 45 and 32 individuals of *Inia geoffrensis*, *I. boliviensis* and *Inia* from the Araguaia River. The 10 microsatellite loci were unlinked, and at Hardy-Weinberg equilibrium within each lineage. In *I. geoffrensis* and *I. boliviensis* all 10 loci were polymorphic, while in the *Inia* from the Araguaia River only eight loci were polymorphic. In *I. geoffrensis*, *I. boliviensis* and the *Inia* from the Araguaia River we observed 12, 10 and 3 alleles in the cytochrome *b* gene, and 9, 6 and 2 alleles in the cytochrome oxidase *c* I gene, respectively.

**Figure 2:**
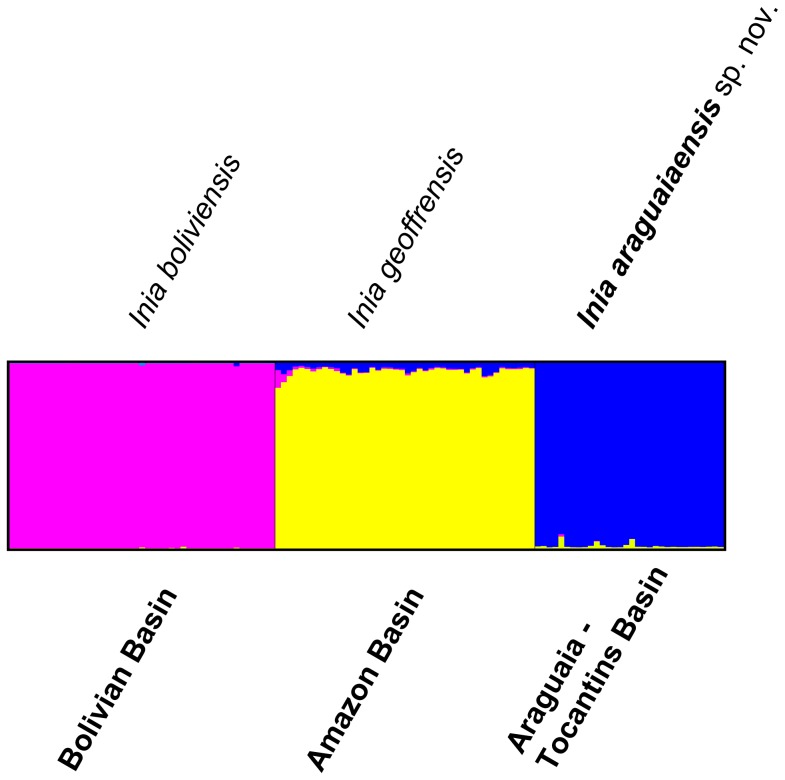
Bayesian inference of population structuring using STRUCTURE 2.3.3 [19], [20]. We analyzed 10 nuclear microsatellite loci sampled from 45 *I. boliviensis*, 44 *I. geoffrensis* and 32 *I. araguaiaensis* sp. nov. individuals. Highest posterior probability is associated with three biological groups; mean LnP(K = 3) = −2041.115.

Analyses conducted with the program STRUCTURE [20], [53] assuming the admixture model, which allows individuals to have ancestors from more than one biological group, and interpreted using Bayes’ Rule [24] identify three groups as the most likely number of biological groups within the genus *Inia* (Fig. 2). Mean posterior probabilities for a given number of biological groups are listed in Table 1. The three biological groups corresponded to the group from the Araguaia River and to the species *I. geoffrensis* and *I. boliviensis*. All but three individuals from the Araguaia River group had a clustering coefficient (*q*) higher than 99%; the remaining three individuals had a *q* value greater than 95%, indicating a lack of genetic admixture of the Araguaia River group with the species *I. geoffrensis* and *I. boliviensis*. Individuals with clustering coefficients larger than 90% are normally considered to have originated from only one biological population (according to STRUCTURE manual), i.e. are non-admixed.

**Table 1:** Mean posterior probabilities of each *a priori* number of biological groups of *Inia* (K) analyzed.

K	Repetitions	Mean LnP(K)	Stdev LnP(K)
1	20	−3124.565000	0.198083
2	20	−2264.810000	0.656867
3	20	−2041.115000	1.433169
4	20	−2041.505000	15.357408
5	20	−2104.215000	36.519573
6	20	−2172.155000	71.411944

**Figure 3:**
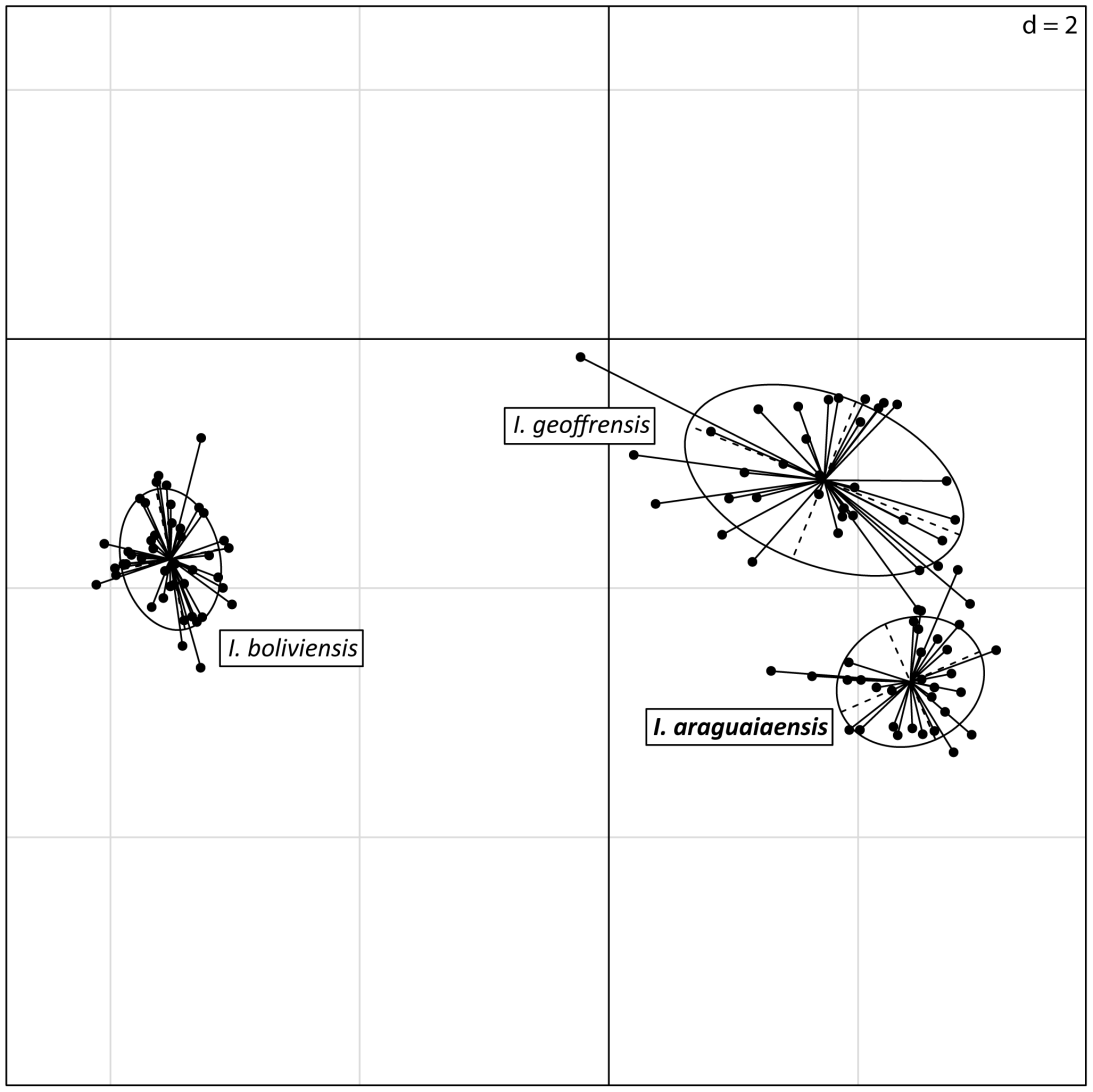
Principal component analysis of the microsatellite data [25] performed in the Ade4 package in the R vers. 2.14.1 statistical program. Principal component axes 1 and 2 are plotted on the X and Y axes, respectively, and ellipses denote 67.53% density contours around each sample group.

**Figure 4:**
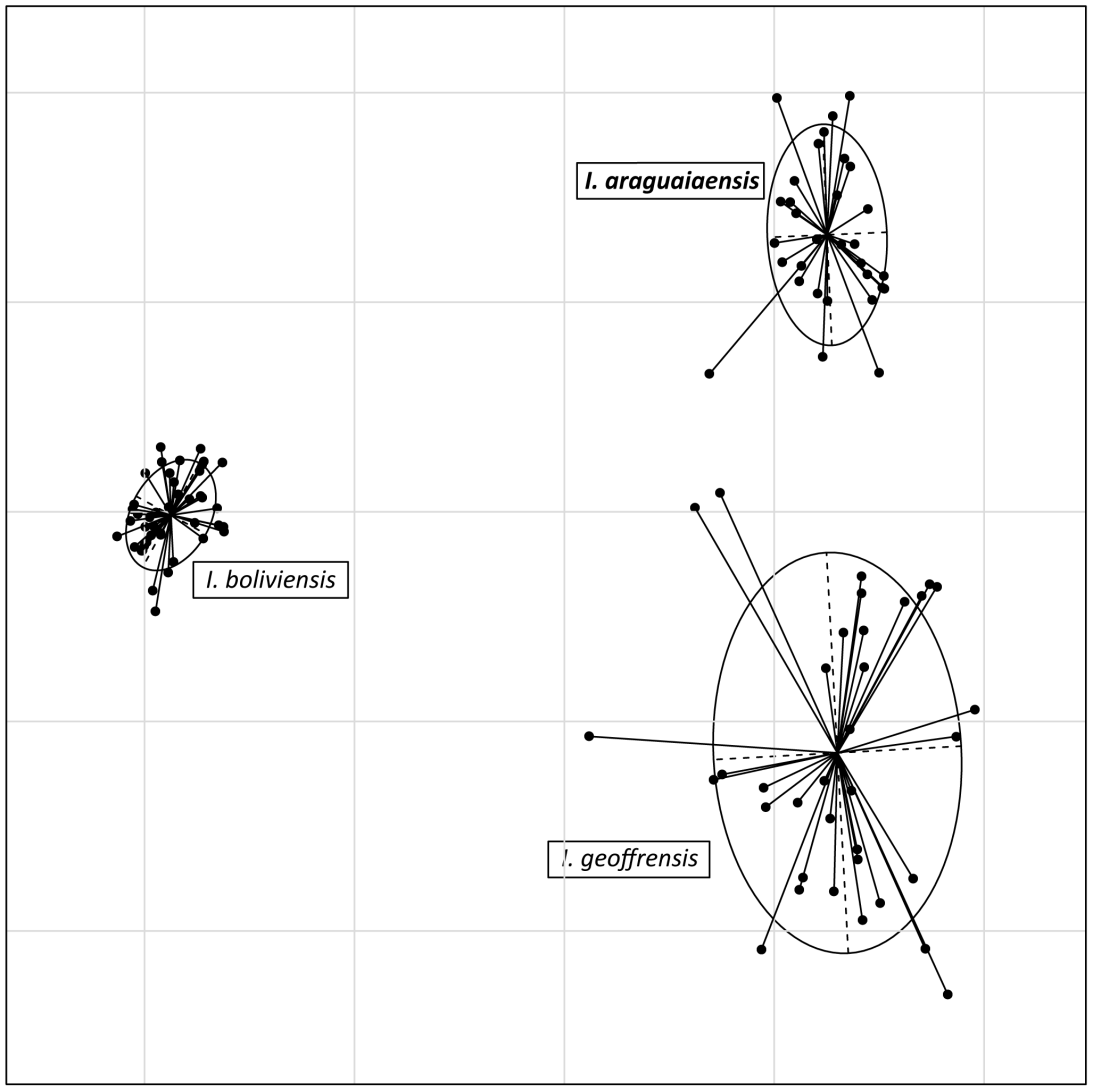
Correspondence analysis of the microsatellite data [25] performed in the Ade4 package in the R vers. 2.14.1 statistical program. Factors 1 and 2 are plotted on the X and Y axes, respectively, and ellipses denote 67.53% density contours around each sample group.

Principal component analysis (PCA) of microsatellite data carried out in the R package Ade4 [25] also indicates the presence of three groups of individuals corresponding to *Inia* individuals from the Araguaia River, and the species *I. geoffrensis* and *I. boliviensis* (Fig. 3). Assuming *a priori* the existence of three groups representing the individuals from the Araguaia River and the species *I. geoffrensis* and *I. boliviensis*, correspondence analysis (COA) unambiguously separates these three entities (Fig. 4).

**Figure 5:**
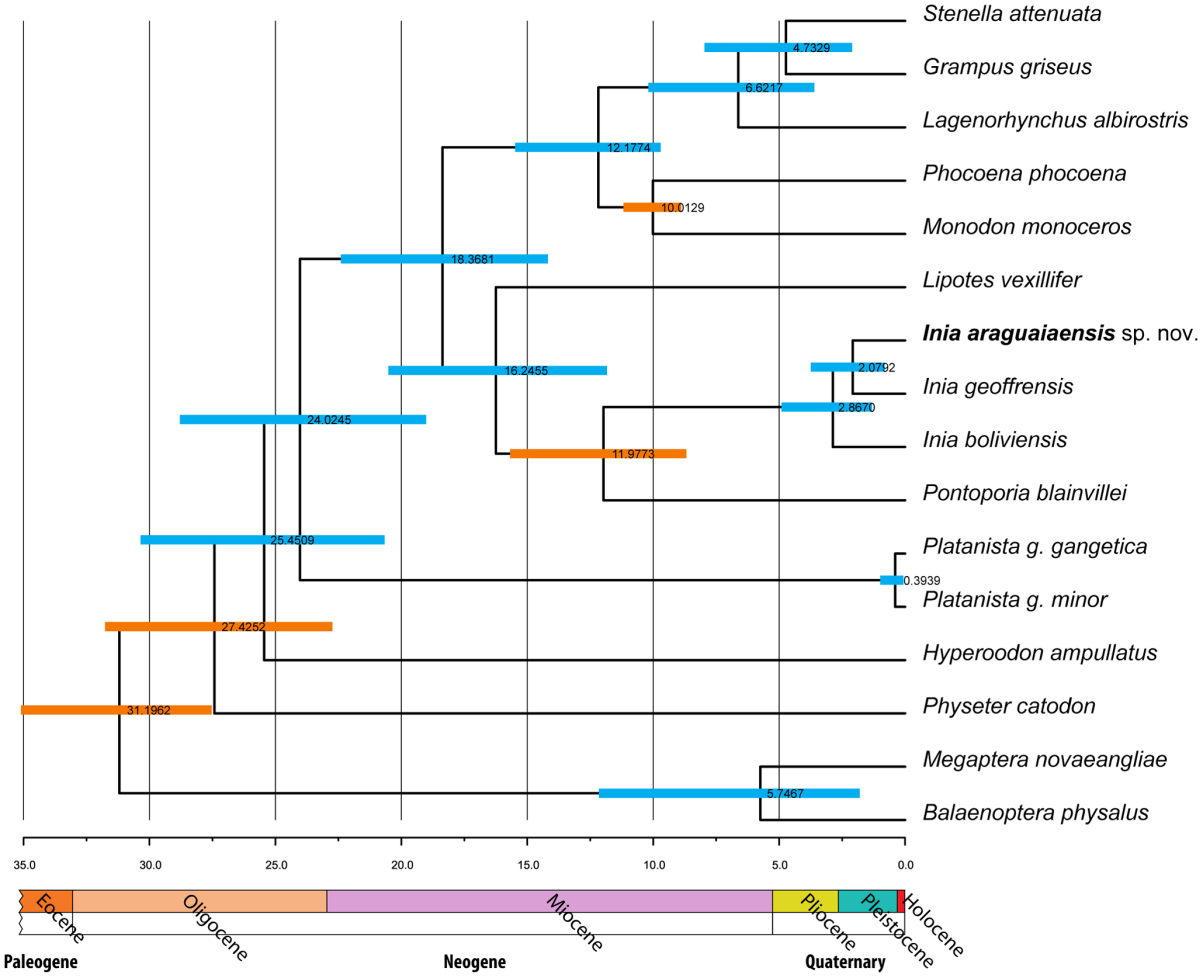
Bayesian phylogenetic analysis and divergence time estimation in BEAST 1.7.1 [45] using the complete mitochondrial DNA cytochrome *b* gene. We used four independent fossil calibration points (indicated as orange bars). Numbers at nodes represent estimates of divergence times with highest posterior probability, while bars around each divergence time estimate represent 95% highest posterior density of the estimate.

The Population Aggregation Analysis [29] of mitochondrial sequence data identified three clusters corresponding to the group from the Araguaia River and to the species *I. geoffrensis* and *I. boliviensis*. The group from the Araguaia River is diagnosable by 28 and 9 apomorphic character states in the mitochondrial cytochrome *b* and the barcode region of the cytochrome oxidase *c* I gene, respectively, while *I. geoffrensis* is diagnosable by 18 apomorphic characters in the cytochrome *b* and 15 in the barcode region of the cytochrome oxidase *c* I gene, and *I. boliviensis* is diagnosable by 21 apomorphic characters in the cytochrome *b* and 9 in the barcode region of the cytochrome oxidase *c* I gene (Table 2). Phylogenetic analyses under both Maximum Parsimony [31] and Bayesian Inference [41] place all individuals of *Inia* from the Araguaia River into a strongly supported sister clade to *I. geoffrensis* (Fig. S1). Furthermore, the estimated divergence of the Araguaia River *Inia* clade from its *I. geoffrensis* sister lineage is 2.08 million years (95% HPD 0.811–3.744 million years) (Fig. 5); converted into a substitution rate, this divergence results in an expected substitution rate of 1.01×10^−8^ substitutions per site per year. The clade formed by *Inia* from the Araguaia basin and *I. geoffrensis* from the Amazonas basin is sister to *I. boliviensis* with an estimated time of divergence of 2.87 mya (95% HPD 1.315-4.903 mya) (Fig. 5).

**Table 2:** 

A – cytochrome *b* diagnostic characters of the three species of Inia			
Character	*I. araguaiaensis*	*I. geoffrensis*	*I. boliviensis*
38	**C**	T	T
45	T	T	C
51	A	A	T
120	C	T	C
138	A	A	G
165	**T**	C	C
177	**C**	T	T
186	**T**	C	C
198	C	T	C
201	T	T	C
210	**T**	C	C
244	T	T	C
258	G	A	G
264	**T**	C	C
282	**C**	T	T
291	T	C	T
306	G	G	A
361	**C**	T	T
365	T	T	C
375	T	C	T
384	**C**	T	T
480	**G**	A	A
555	**T**	C	C
574	C	T	C
576	A	A	G
582	**C**	T	T
585	**A**	G	C
589	C	C	T
633	**T**	C	C
654	C	C	T
657	A	A	T
690	A	A	G
693	**G**	A	A
699	**C**	T	T
702	A	A	G
724	T	T	C
745	**T**	C	C
753	**G**	A	A
770	**T**	C	C
771	C	C	T
792	C	C	T
801	**T**	C	C
807	G	G	A
813	G	G	A
816	**A**	G	G
819	T	C	T
825	G	G	A
850	**G**	A	A
867	**A**	G	G
870	**G**	A	A
873	A	G/C	A
891	A	A	G
909	C	T	C
924	T	C	T
984	A	T	A/G
1008	A	C	A
1020	**T**	C	C
1041	**T**	C	C
1047	C	C	T
1048	A	G	A
1066	**C**	A	A
1080	T	C	T
1089	A	G	A
1092	**A**	G	G
1113	T	C	T
**B – cytochrome oxidase I barcode region diagnostic characters of the three species of Inia**			
**Character**	***I. araguaiaensis***	***I. geoffrensis***	***I. boliviensis***
24	A	A	G
30	C	C	T
51	G	A	G
60	T	C	T
87	**G**	A	A
99	T	C	T
125	G	A	G
159	T	T	C
174	**A**	G	G
177	T	C	T
180	A	A	G
231	A	G	A
241	**A**	G	G
243	**A**	G	G
258	**A**	G	G
270	A	G	A
276	T	T	C
279	G	A	G
288	T	T	C
291	G	G	A
309	C	C	T
327	C	T	C
353	C	T	C
374	**G**	A	A
381	A	G	A
387	**A**	G	G
395	**G**	A	A
417	G	A	G
426	G	G	A
435	A	A	G
498	T	T	C
507	A	A	G
519	G	A	G
531	C	T	C
552	A	A	G
558	**T**	C	C
582	A	T	A

Species level diagnostic characters observed in the mitochondrial cytochrome *b* gene region (**A**), *Inia araguaiaensis* sp. nov. is diagnosable by 27 molecular synapomorphies, *I. boliviensis* by 21 molecular synapomorphies and *I. geoffrensis* by 18 molecular synapomorphies. Species level diagnostic characters in the cytochrome oxidase I DNA barcode region (**B**), *I. araguaiaensis* sp. nov. is diagnosable by 9 molecular synapomorphies, *I. boliviensis* by 15 molecular synapomorphies and *I. geoffrensis* by 13 molecular synapomorphies. First line indicates position of the character within the cytochrome *b* gene and the cytochrome oxidase I DNA barcode region.

Diagnostic characters of *I. araguaiaensis* are in bold.

Analysis of osteological and morphometric data indicates a number of differences between the *Inia* individuals from the Araguaia River and *I. geoffrensis* and *I. boliviensis*. In general the width of the braincase across the parietals as well as the greatest width of the maxillaries and the greatest parietal width are larger when compared with *I. geoffrensis* and *I. boliviensis*. Specifically diagnostic differences between the Araguaia *Inia* and *I. geoffrensis* are in the width of the braincase across the parietals of females (range: 9.6 to 11.5 cm, mean: 10.6 cm, n = 2 *vs*. range: 11.6 to 13.5 cm, mean: 12.4 cm, n = 9). Principal differences between the Araguaia *Inia* and *I. boliviensis* are in the number of teeth per hemimandible (24–28) when compared with *I. boliviensis* (31–35); larger greatest parietal width in males (range: 8.1 to 9.2 cm, mean: 8.7 cm, n = 2 *vs*. range: 5.2 to 7.6 cm, mean: 6.7 cm, n = 3); and larger greatest width of maxillaries in females (range: 11.3 to 11.8 cm, mean: 11.6 cm, n = 2 *vs*. range: 9.3 to 10.4 cm, mean: 9.8 cm, n = 5).

There is, therefore, strong evidence that *Inia* individuals from the Araguaia River represent a biological group distinct from *I. geoffrensis* and *I. boliviensis*. They also form a lineage with an evolutionary trajectory independent of *I. geoffrensis* and *I. bolivensis* since the early Pleistocene. Furthermore, there exist morphological, albeit subtle, differences between the *Inia* individuals from the Araguaia River and individuals of *I. geoffrensis* and *I. boliviensis*. Therefore we recognize and designate the population of *Inia* inhabiting the Araguaia River as a new species.

*Cetacea* Brisson, 1762.

*Odontoceti* Flower, 1867.

*Iniidae* Gray, 1846.

*Inia* d’Orbigny, 1834.

### 

#### *Inia araguaiaensis* sp. Nov

Hrbek, Farias, Dutra and da Silva 2014 urn:lsid:zoobank.org:act:399CD69C-6452-46C4-BDA9-2416409B50A7.

#### Etymology

The specific name refers to the geographic area where the new species is currently known to occur.

#### Suggested common name

We propose the common names “Boto-do-Araguaia” and “Araguaian boto” for this species. Boto is the common regional and international name of species of *Inia*, and Araguaia refers to the geographic distribution of this species.

#### Holotype

**Figure 6:**
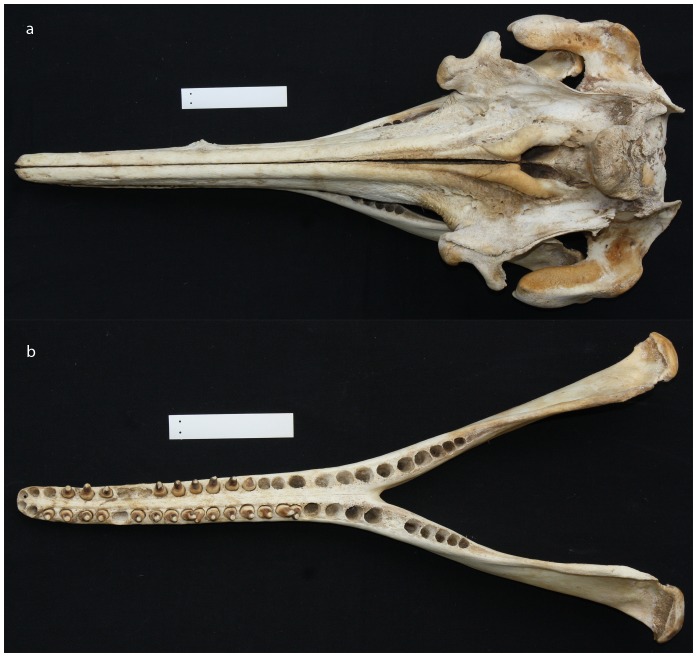
The cranium (6a) and the mandible (6b) of the holotype of *Inia araguaiaensis* sp. nov.; 10 cm ruler shown.

INPA MA 144 (Fig. 6); Adult male, skeleton partially complete (skull, mandibles, total of 68 teeth, 2 tympanic bulla, 1 sternum, hyoid apparatus [1 basyhyal and 2 stylohyal], a total of 37 vertebrae [7 cervical, 11 thoracic, 3 lumbar, 16 caudal], 8 chevrons, 12 pair of ribs, 1 floating rib) and fragments of skin preserved in alcohol deposited at the Mammal Collection of the National Institute for Amazonian Research (INPA), Manaus, Amazonas, Brazil. The specimen was collected near the entrance of Lake Jurumirim (15° 10′ 19.65″ S; 51° 38′ 48.30″ W), Araguaia River, state of Goiás on 21 September 2009 by Claryana Costa Araújo. The type was found floating dead, in an early stage of decomposition.

#### Paratypes

INPA MA 145 and INPA MA 146; skulls and mandibles of one adult male (INPA MA 145) with a total of 87 teeth available and one pregnant female (INPA MA 146) with a total of 72 teeth available. These specimens were both collected on 19 August 2010, about 10 km apart, below and above the entrance of Jurumirim Lake, on the Araguaia River. Collectors were V.M.F. da Silva and N. Dutra, and the material was deposited at the Mammal Collection of the National Institute for Amazonian Research (INPA), Manaus, Amazonas, Brazil, together with fragments of skin preserved in alcohol.

#### Distribution

*Inia araguaiaensis* sp. nov. occurs in the central region of Brazil, in the Araguaia River basin (Fig. 1). We recorded it in the lower and middle Araguaia River from Barra do Garças (15° 54′ 02.8″S; 52° 16′ 58.7″W) to the Santa Isabel rapids (06° 09′ 01.1″S; 48° 22′ 14.7″W), and in several tributaries such as Vermelho, Peixe, Crixás-Açú and Água Limpa Rivers, and dos Tigres and Rico Lakes, all in the state of Goiás, and Lake Montaria in the state of Mato Grosso. The species is likely present in the Tocantins River [54], and potentially in the whole Araguaia-Tocantins River Basin.

#### Diagnosis

*Inia araguaiaensis* sp. nov. differs from all its congeners by having the following unique characters: 28 diagnostic sites (apomorphies) in the complete mitochondrial DNA cytochrome *b* gene; 9 diagnostic sites in the cytochrome oxidase *c* I DNA barcode region; a reduced number of teeth per hemimandible (24–28) when compared with *I. boliviensis* (31–35); narrower braincase across parietals of females (range: 9.6 to 11.5 cm, mean: 10.6 cm, n = 2) compared to *I. geoffrensis* females (range: 11.6 to 13.5 cm, mean: 12.4 cm, n = 9); larger greatest parietal width in males (range: 8.1 to 9.2 cm, mean: 8.7 cm, n = 2) compared to *I. boliviensis* males (range: 5.2 to 7.6 cm, mean: 6.7 cm, n = 3); and larger greatest width of maxillaries in females (range: 11.3 to 11.8 cm, mean: 11.6 cm, n = 2) compared to *I. boliviensis* females (range: 9.3 to 10.4 cm, mean: 9.8 cm, n = 5).

#### Description

*Inia araguaiaensis* sp. nov. shows rather subtle morphological differences when compared to *I. geoffrensis* and more accentuated differences when compared to *I. boliviensis*. All species of the genus *Inia* show two distinct types of teeth, but *I. araguaiaensis* sp. nov. possesses 24–28 teeth per hemimandible compared to the 25–29 in *I. geoffrensis* and 31–35 in *I. boliviensis*. The 19–21 front pairs of teeth in both upper and lower jaws are conical, but the posterior 6–8 pairs (cheek teeth) have one pointed cusp extending from the crown towards the labial side and a depression on the lingual side. The width of the brain-case across parietals as well as the greatest width of maxillaries and the greatest parietal width are larger when compared with the two other species. *Inia araguaiaensis* sp. nov. is the sister species to *I. geoffrensis*, sharing their most recent common ancestor 2.08 mya. It forms a biological group distinct from *I. geoffrensis* and *I. boliviensis* and there is strong indication of reproductive isolation from these species.

## Discussion

All species concepts applicable to sexually reproducing organisms that conceive of species as an outcome of an evolutionary process implicitly or explicitly assume that species are groups of individuals that can freely exchange genetic information with each other, but are unable to do so with individuals of other species. For example, the Biological Species Concept (BSC) focuses on ongoing patterns of reproduction, using actual or potential reproduction among individuals of the species as a criterion for identifying species limits [18]. On the other hand, the Phylogenetic Species Concept (PSC) focuses on historical patterns of reproduction that would maintain autonomous evolutionary lineages [26]–[28]. Our analyses identify *I. araguaiaensis* sp. nov. as both an evolutionary lineage and a group of actually interbreeding individuals, satisfying the criteria necessary for the recognition of this taxon as a species under the Biological as well as Phylogenetic Species Concepts.

Analyses conducted with the program STRUCTURE [19], [20] and interpreted using Bayes’ Rule [24] identify three groups as the most likely number of biological groups within the genus *Inia* (Fig. 2). These biological groups represent *I. araguaiaensis* sp. nov., *I. geoffrensis* and *I. boliviensis*. The probability that only two biological groups exist, and that *I. araguaiaensis* sp. nov. individuals belong to the same biological group as *I. geoffrensis* is 4.22×10^−98^. Allowing for individuals to have ancestors from more than one biological group results in the inference that individuals of *I. araguaiaensis* sp. nov. do not have admixed ancestry. This does not necessarily mean that there is no gene flow or that there has been no recent historical gene flow between *I. araguaiaensis* sp. nov. and either *I. geoffrensis* or *I. boliviensis*, but the data do indicate that all three species represent distinct biological populations characterized by a shared system of mating among members of each species (sensu Templeton [55]), but not among species. Evolutionary distinctness of *I. araguaiaensis* sp. nov., *I. geoffrensis* and *I. boliviensis* is also manifested by diagnostic alleles, distinct allelic frequencies and allelic combinations of each species as observed in the PCA (Fig. 3). Assuming the existence of the three species of *Inia a priori*, the three species are clearly separable in multivariate allelic space (Fig. 4).

*Inia araguaiaensis* sp. nov., *I. geoffrensis* and *I. boliviensis* are also diagnosable as biological entities evolutionarily independent of each other, i.e. lineages characterized by independent evolutionary trajectories. As such, each species is diagnosable by a series of unique characters. These characters are both molecular (Table 2) and morphological (see Diagnosis above). However, due to morphological conservatism, the majority of the diagnostic characters are molecular.

The three species have a long history of evolutionary independence with strongly supported chronological order of speciation events (Figs. 2 & 3). The earliest species-level divergence resulted in the separation of *I. boliviensis* from the lineage leading to *I. geoffrensis* and *I. araguaiaensis* sp. nov. in the late Pliocene (Fig. 5). This divergence was followed shortly by the divergence of *I. geoffrensis* from *I. araguaiaensis* sp. nov. in the early Pleistocene (Fig. 5). Both divergences are associated with major geological events in the Amazon basin.

With highest probability *I. araguaiaensis* sp. nov. diverged from its *I. geoffrensis* sister lineage 2.08 mya (95% HPD 0.811–3.744 mya) (Fig. 5). This divergence time corresponds to the last time that the Araguaia-Tocantins basin discharged directly into the Amazon River at the Pliocene/Pleistocene boundary [2]. At the time of the Rossetti and Valeriano study [2], the Pliocene included the Gelasian stage, which lasted from 2.588 to 1.806 mya [56], and therefore the authors estimated an isolation of the Araguaia-Tocantins basin at 1.8 mya. The estimated isolation of the Araguaia-Tocantins basin from the Amazon basin 1.8 mya corresponds closely to the estimated divergence of *I. araguaiaensis* sp. nov. from the Araguaia basin and *I. geoffrensis* from the Amazon basin at 2.08 mya (Fig. 5), leading us to the conclusion that speciation was vicariant.

Our results also clearly show that *I. boliviensis* is a distinct species. *Inia boliviensis* is an independent biological population (Figs. 2 & 3), and the estimated time of divergence of *I. boliviensis* from its sister clade is 2.87 mya (95% HPD 1.315–4.903 mya) (Fig. 5). This divergence estimate is broadly compatible with the uplift of the Fitzcarrald Arch which commenced to isolate the Bolivian sub-basin from the Amazon basin no earlier than 4 mya [57], [58]. The observed divergence between *I. boliviensis* and its sister clade has, therefore, likely been driven by the uplift of the Fitzcarrald Arch, and has lead to vicariant speciation. Similar signatures of vicariant diversification can be observed in other Amazonian aquatic vertebrate groups such as the piranhas of the genera *Pygocentrus* and *Serrasalmus* [59] and peacock basses of the genus *Cichla* [60].

Currently much of the scientific community [61], [62], including the IUCN (http://www.iucnredlist.org/details/10831/0), consider *I. boliviensis* to be a subspecies of *I. geoffrensis*. However, in 2012 the Ad-Hoc Committee on Taxonomy of the Society for Marine Mammalogy (http://www.marinemammalscience.org/) started considering *I. boliviensis* a full species citing the control region mtDNA data [4] and nuclear introns data [63]. We confirm this taxonomic reassessment and demonstrate that *I. boliviensis* is diagnosable under the biological species (Figs. 2 & 3) as well as the phylogenetic species (Table 1 and Fig. 5) criteria.

Similar to the taxonomic uncertainty involving *I. boliviensis*, one may question whether or not *I. araguaiaensis* sp. nov. represents a valid taxon. The observed phylogenetic and mating-system patterns could potentially represent intraspecific variation in a strongly genetically structured species. We reject this notion in favor of the existence of a new species in the Araguaia River basin on the grounds that *Inia araguaiaensis* sp. nov. 1) has no apparent actual or recent history of admixture with other *Inia* species, 2) has a large number of diagnostic characters that unambiguously discriminate it from other *Inia* species, and 3) diverged from its sister taxon *I. geoffrensis* with highest probability 2.08 million years ago, but no less than 0.811 million years ago. This divergence time exceeds most sister-taxon divergence-time estimates in the closely related family Delphinidae [44], [64]. Therefore, all the presented data corroborate our interpretation that *I. araguaiaensis* sp. nov. is an evolutionary species, i.e. a lineage with an independent evolutionary trajectory.

### Conservation

True river dolphins are some of the rarest and most endangered of all vertebrates comprising relict evolutionary lineages of high taxonomic distinctness [65], [66]. Of the four extant species listed by the IUCN, three are listed in one of the IUCN Red List’s threat categories (VU – *Pontoporia blainvillei*, EN – *Platanista gangetica*, CR – *Lipotes vexillifer*) while *Inia geoffrensis* is DD. Although listed as critically endangered by the IUCN, the Chinese baiji (*Lipotes vexillifer*), became extinct between 2004 and 2006 [67]. The description of *I. araguaiaensis* represents the first description of a new true river dolphin since 1918 when the now extinct *L. vexillifer* was described. *Lipotes vexillifer* was also the first river dolphin, and probably the first cetacean, to become extinct as a result of anthropogenic activities [67]–[69]. The conservation status of *I. araguaiaensis* has not been assessed, but merits attention. *Inia araguaiaensis* appears to have low levels of genetic diversity, and also has a restricted distribution in what we estimate to be an approximately 1500 km stretch of the Araguaia River with additional fragmented habitat in the Tocantins River basin [54]. The number of individuals is also probably low. In an environmental impact study conducted for the licensing of the Santa Isabel hydroelectric project on the lower Araguaia River, a total of 122 dolphins were seen over a 12 week period in 60 km survey stretch of the river [70]. Assuming an average width of 0.5 km [71], and assuming that no animal was seen more than once, this would represent approximately 1525 individuals in the Araguaia River basin. Araújo and da Silva [72] estimated, depending on the transect type used, 0.80 to 0.98 indiv/km^2^ in the Araguaia River suggesting a population of 600 to 735 individuals. Araújo [71], assuming that *I. araguaiaensis* occurs throughout the total 2110 km length of the Araguaia River estimated a total of 1033 individuals Extrapolating from the highest *I. geoffrensis* density of 1.3 indiv/km^2^ observed in non-protected areas of the Amazon basin (the Napo River in Ecuador) [73], 975 individual could occur in the Araguaia River basin. Populations of the middle and upper Tocantins River are fragmented by six hydroelectric dams, not including the Tucurui dam, and are likely to have very few individuals. Since the 1960’s the Araguaia River basin has been experiencing significant anthropogenic pressure via agricultural and ranching activities, and the construction of hydroelectric dams, all of which have had negative effects on many biotic and abiotic aspects of the functioning of the Araguaia River ecosystem [74] which *I. araguaiaensis* inhabits. Similarly *I. araguaiaensis* in the Tocantins River is affected by agricultural and industrial development, and is fragmented into isolated populations by hydroelectric dams [54]. Interpreting these data using IUCN criteria, *Inia araguaiaensis* should minimally be considered vulnerable (VU Red List category).

## Supporting Information

Figure S1 **Bayesian phylogenetic analysis carried out in MrBayes 3.1.2** [**32**] **.** Numbers above nodes represent posterior probabilities. We used all 45 *I. boliviensis*, 44 *I. geoffrensis* and 32 *I. araguaiaensis* sp. nov. individuals in this analysis. Intraspecific relationships were collapsed and represented as a triangle with the depth of the triangle representing intraspecific divergence, and height of the triangle number of individuals. Maximum parsimony topology is identical.
(TIF)
