# Supplementary figures and images for: A New Species of River Dolphin from Brazil or: How Little Do We Know Our Biodiversity
Source: PLoS One. 2014 Jan 22;9(1):e83623. doi: 10.1371/journal.pone.0083623 (PMC3898917; doi:10.1371/journal.pone.0083623)

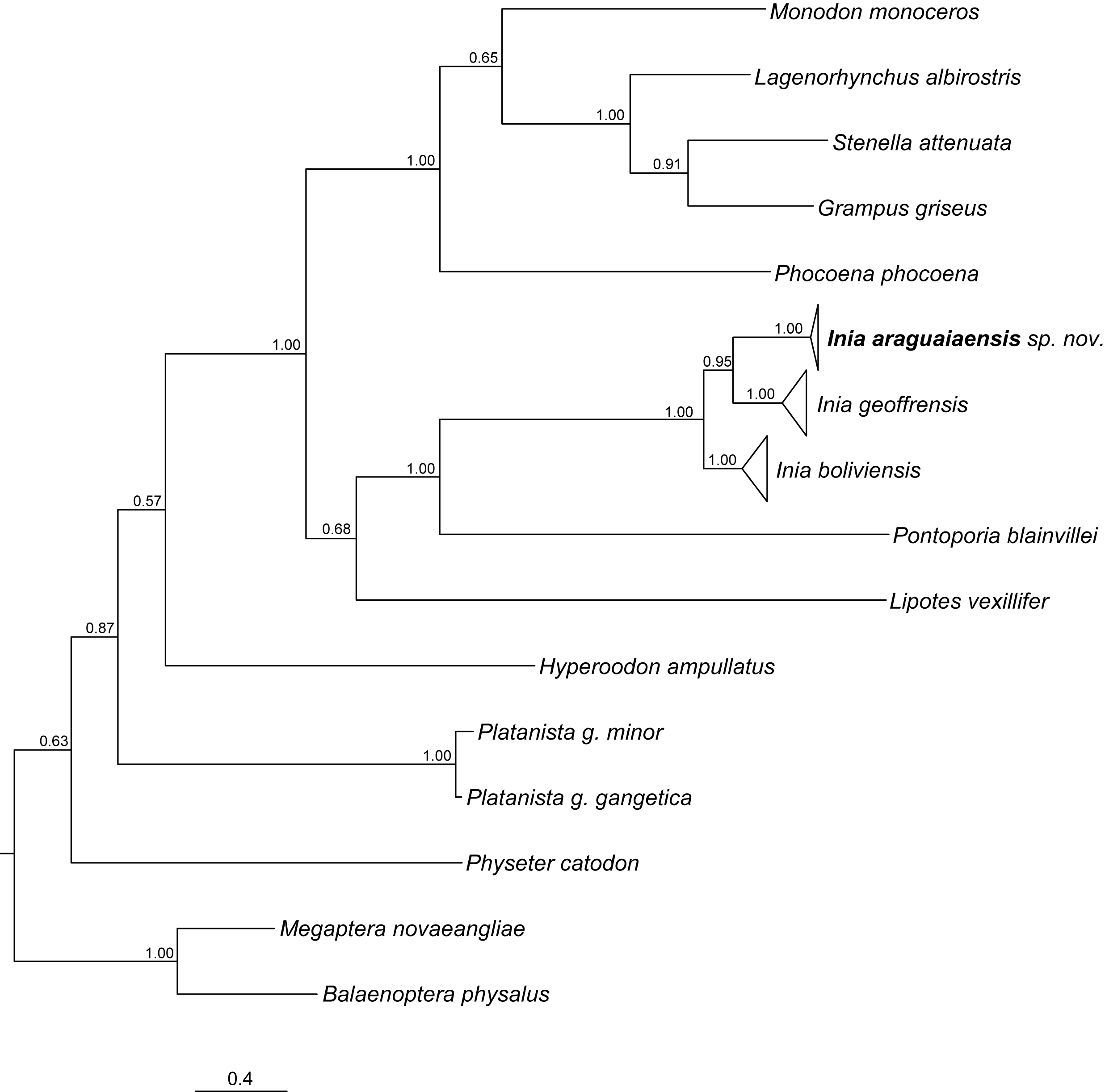

Supplement: Figure S1 — Bayesian phylogenetic analysis carried out in MrBayes 3.1.2 [32] . Numbers above nodes represent posterior probabilities. We used all 45 I. boliviensis, 44 I. geoffrensis and 32 I. araguaiaensis sp. nov. individuals in this analysis. Intraspecific relationships were collapsed and represented as a triangle with the depth of the triangle representing intraspecific divergence, and height of the triangle number of individuals. Maximum parsimony topology is identical. (TIF) [file pone.0083623.s001.tif]
